# Rush@Home: Home-Based Primary Care Focused on Underserved Communities and Health Equity

**DOI:** 10.1093/geroni/igab046.1031

**Published:** 2021-12-17

**Authors:** Robyn Golden, Alexander Rackman, Elizabeth Davis, Leticia Santana, Walter Rosenberg

**Affiliations:** Rush University Medical Center, Chicago, Illinois, United States

## Abstract

Homebound patients are often medically complex and are among those in greatest need of care and services. This is especially true for those that reside in underserved communities, where they face the added risk stemming from scarce community resources. Often these patients are only able to access health care for emergencies, which is ineffective and high cost. Rush@Home is a home-based primary care program that exemplifies the Age-Friendly Health System mission with a focus on the 4Ms, incorporating navigation and social work. Patients reflect the West Side of Chicago, with 80% of patients identifying as Black and/or Latino. During the first two years, Rush@Home demonstrated better care at a lower cost with readmission rates decreased by 11.8%, hospitalizations by 17.5%, length of stay by 8.7%, ED visits by 17.9%, and missed appointments by 72%. This presentation will highlight outcomes and discuss key issues in home-based primary care.

